# Effects of gestational diabetes mellitus on risk of adverse maternal outcomes: a prospective cohort study in Northwest Ethiopia

**DOI:** 10.1186/s12884-020-2759-8

**Published:** 2020-02-03

**Authors:** Achenef Asmamaw Muche, Oladapo O. Olayemi, Yigzaw Kebede Gete

**Affiliations:** 1 Department of Obstetrics and Gynecology, Pan African University Life and Earth Sciences Institute, College of Medicine, University of Ibadan, Ibadan, Nigeria; 2 Department of Epidemiology and Biostatistics, Institute of Public Health, University of Gondar, Gondar, Ethiopia; 3 Department of Obstetrics and Gynecology, College of Medicine, University College Hospital, University of Ibadan, Ibadan, Nigeria

**Keywords:** Gestational diabetes mellitus, adverse maternal outcome, pregnancy induced hypertension, premature rupture of membranes, antepartum hemorrhage, postpartum hemorrhage

## Abstract

**Background:**

Gestational diabetes mellitus is a leading medical condition woman encounter during pregnancy with serious short- and long-term consequences for maternal morbidity. However, limited evidence was available on potential impacts of gestational diabetes mellitus using updated international diagnostic criteria on adverse maternal outcomes. Therefore, this study aimed to assess the effects of gestational diabetes mellitus on the risk of adverse maternal outcomes in Northwest Ethiopia.

**Methods:**

A prospective cohort study was conducted among pregnant women followed from pregnancy to delivery. Gestational diabetes mellitus status was determined by using a two-hour 75 g oral glucose tolerance test and based on updated international diagnostic criteria. Multivariable log-binomial model was used to examine the effects of gestational diabetes mellitus on the risk of adverse maternal outcomes.

**Results:**

A total of 694 women completed the follow-up and included in the analysis. Women with gestational diabetes mellitus had a higher risk of composite adverse maternal outcome (ARR=1.58, 95% CI: 1.22, 2.04), caesarean delivery (ARR=1.67; 95%: 1.15, 2.44), pregnancy induced hypertension (ARR= 3.32; 95%: 1.55, 7.11), premature rupture of membranes (ARR= 1.83; 95%: 1.02, 3.27), antepartum hemorrhage (ARR= 2.10; 95%: 1.11, 3.98) and postpartum hemorrhage (ARR= 4.85; 95%:2.28, 10.30) compared to women without gestational diabetes mellitus.

**Conclusions:**

Gestational diabetes mellitus increased the risk of adverse maternal outcomes. This implies that maternal care and intervention strategies relating to women with gestational diabetes mellitus should be strengthened.

## Background

Gestational diabetes mellitus (GDM) is defined as “hyperglycemia first detected during pregnancy that is clearly not preexisting or overt diabetes” [1]. It is believed to be the drastically increased prevalence of GDM had a negative impacts on various short- and long-term maternal and neonatal adverse outcomes [2, 3].

Gestational diabetes mellitus has been associated with an increased risk for pregnancy induced hypertension (PIH) with relative risk ranges from 1.4 to 4.15 [4–9] although some studies suggest that the relation between PIH and GDM is not well understood [10, 11]. It also increases the rate of cesarean delivery by up to 57.4% and has a greater impact in cases of obesity and/or previous history of cesarean section [12–18]. The risk of induction of labor ranges from 33–38% [13, 18–20], premature rupture of membranes (PROM) [9, 21–23], antepartum hemorrhage (APH) [24], and postpartum hemorrhage (PPH) were associated with GDM [2, 23]. To the contrary, other studies showed the absence of significant association between GDM and the severity of the risk for PPH [25] and PIH [11, 26]. Thus, evidence is inconclusive; the extent to which the observed associations were caused by maternal factors or were confined to their poor socioeconomic conditions, behavioral or lifestyle parameters and lack of health care services was still debatable at the moment.

While the adverse maternal outcomes of women with GDM can be improved by proper antenatal care and positive lifestyle changes [27, 28], the risk for adverse outcomes drastically increased as result of increase in maternal glucose level in the second or third trimester, even within ranges previously considered normal for pregnancy [29, 30].

Though, the consequences of GDM on adverse maternal outcomes have been recognized in developed countries with different controversies, there are limited data on the effect of GDM in sub-Saharan Africa (SSA) countries where emphasis to it is low. We have also noted that the aforementioned studies used different diagnostic criteria and thresholds, mainly the old GDM diagnostic criteria that could have either under or overestimated the adverse maternal outcomes caused by GDM. Thus, the overall risk of adverse maternal outcomes on women with GDM remains unclear because studies based on the current GDM diagnostic criteria are limited. Therefore, this study aimed to examine the effects of GDM on adverse maternal outcomes using the current updated diagnostic criteria. In addition, the study considered whether the relationship between GDM and adverse maternal outcomes was mediated by lifestyle parameters, such as levels of physical activity and dietary diversity and the extent was affected by the presence of antenatal depression.

## Methods and Materials

### Study area and period

The study was conducted at selected public health facilities of Gondar town (the University of Gondar Comprehensive Specialized Hospital (UoGCSH) and Gondar, Woleka, Maraki, Azezo Health Centers) from March 30, 2018 to March 26, 2019. Gondar town is located in Northwest Ethiopia 747 km from Addis Ababa (the capital of Ethiopia) and 170 km from Bahirdar (the capital of the Amhara regional state). According to the 2014 Central Statistical Agency (CSA) population projection, the town had a total population of 306, 246, of whom 149, 970 were men and 156, 276 women [31]. The area is predominantly urban and the town has one public referral hospital, eight health centers, and 15 private clinics.

### Study design and population

A prospective cohort study was conducted on a group of pregnant women recruited at the ANC clinics of the selected health facilities of Gondar town and followed from pregnancy to delivery. Pregnant women were recruited and followed if they were aged 18 years and above, had gestational age of 20 - 23^+6^ weeks with a singleton pregnancy, was permanent resident in the study area, were willing to take routine ANC services and had planned to deliver at one of the selected facilities of the town. We excluded women who had pre-existing or overt diabetes or other medical illness and chronic diseases or were on medication that might affect their glucose metabolism (steroids, β-adrenergic agonists, anti-psychotic drugs), and/or severely ill at commencement.

### Sample size and sampling procedure

Sample size was estimated using Epi Info 7 software [32] with the following parameters: confidence level of 95% (2-sided), power of 80 %, exposed to non-exposed ratio of 1:4, prevalence of adverse maternal outcome (preeclampsia) in a non-exposed group (non GDM) of 8.7% and exposed group (GDM) of 27.7% [9]. This study also considered design effect of 2, and lost follow-up and non-response rate of 15%. The minimum sample size required for the study was 476, of which 97 were exposed (GDM) and 386 non-exposed women (non-GDM). Initially, for the baseline survey, about 1,110 study participants were recruited at the beginning of the study and a total of 1027 participants completed the GDM screening enrolled for this cohort study to increase the power of the study. A detail of the study sample is provided elsewhere [33].

### Data collection procedure

A pre-tested structured questionnaire prepared in English and translated to Amharic (national and local language) and retranslated to English by public health and language expert was used to collect data during baseline survey. Data also abstracted using checklist from the medical records of pregnant women who gave birth. Moreover, validated tools were implemented to assess dietary diversity [34], physical activities [35], and antenatal depression [36].

Both primary and secondary data (chart review) were used. Data on maternal and socio-demographic variables (age, residence, marital status, level of education, occupational status, average monthly income, last normal menstrual period (LNMP), previous pregnancy complications, medical history), behavioral factors (exposure to alcohol use and coffee intake), lifestyle parameters (dietary diversity and physical activity) and antenatal depression status were collected by midwives in face to face interview during baseline survey. Blood pressure (BP) and mid-upper arm circumference (MUAC) were also measured.

Data on antepartum (gestational age (early fetal ultrasound result), parity, gravidity, hemoglobin level, blood pressure, urine analysis, APH, co-existing obstetric/medical diseases, complications of pregnancy, sonographic result), intrapartum (duration of labor, types of labor (spontaneous or induced), mode of delivery, and premature rupture of membranes) and postpartum (PPH) were retrieved from medical records and focused antenatal care charts (integrated antenatal, labor, delivery and postnatal care cards) of the pregnant women and documented in the checklist prepared for the purpose.

Universal screening for GDM using a two-hour 75 g oral glucose tolerance test (OGTT) was performed for all pregnant women at 24-28 weeks of gestational age. Besides, for women who had negative result at regular test (24-28 weeks of gestational age) and had at least one type of risk factors for GDM (pre pregnancy BMI ≥ 30 Kg/m^2^, MUAC ≥ 28 cm, age ≥ 35 years, previous macrosomia, glycosuria, history of GDM, family history of diabetes, previous poor pregnancy outcome or developed pregnancy-related complications) were repeated the test at 32-36 weeks. The tests were done directly at the respective health facilities of the participants by capillary glucose testing, using a standard plasma-calibrated glucometer (HemoCue Glucose B-201+ (A¨ngelholm AB, Sweden)). This procedure adhered to the latest recommendations of the International Federation of Gynecology and Obstetrics (FIGO) initiative on GDM diagnosis for settings where a close-by laboratory or facilities for proper storage and transport of blood samples to a distant laboratory are not available [37].

Pregnant women were categorized based on their GDM status and included in this prospective study. All women received follow up care at their respective health facilities under the standard protocol of GDM diagnosis, management, and care services or the health facility’s routine protocol. Participants diagnosed with GDM were immediately referred (linked) to health providers who were experts in their respective public health facilities to get possible management and treatment options. Follow-ups were assured through the health centers and UoGCSH in close collaboration with the experts and data collectors. Lastly, the medical records of pregnant women who gave birth were reviewed and information related to maternal outcomes were documented.

### Study variables

#### Outcome variables

Composite adverse maternal outcome was defined as the occurrence of one or more of the following: cesarean delivery, PIH, induced labor, PROM, APH, and/or PPH. Pregnancy-induced hypertension (PIH) is defined as systolic blood pressure ≥ 140 mmHg and/or diastolic blood pressure ≥ 90 mmHg after 20 weeks of pregnancy [38]. Cesarean delivery is an operative technique by which a fetus is delivered through abdominal and uterine incision [39]. Induction of labor is defined as the process of artificially stimulating the uterus to start labor [40]. Premature rupture of membranes (PROM) refers to a patient who is beyond 37 weeks of gestation and has presented with rupture of membranes (ROM) prior to the onset of labor [40]. Antepartum haemorrhage (APH) is defined as bleeding from or in to the genital tract, occurring during second or third trimesters of pregnancy and prior to the birth of the baby [41]. Postpartum haemorrhage (PPH) is defined as a blood loss of 500 ml or more within 24 hours after birth [42].

#### Primary exposure variable

The primary exposure variable for this study was GDM. Its diagnosis was made by using the 2017 American Diabetes Association (ADA) [43] or the 2013 World Health Organization (WHO) [44] or modified International Association of the Diabetes and Pregnancy Study Groups (IADPSG) [45] diagnostic criteria. The diagnosis of GDM is made when one or more of the values of plasma glucose level was met (fasting: ≥ 92 mg/dL, 1 h: ≥180 mg/dL; 2 h: ≥ 153 mg/dL).

#### Covariates

The following comprise independent variables that are theorized to be non-causal risk factors for adverse maternal outcomes. Some variables are also associated with GDM and are included in this study as confounding variables.

Maternal age was categorized as (< 25, 25–29, 30–34, and ≥ 35 years); marital status as (married, single, divorced or widowed); education as (no formal education, primary, secondary and above); employment status as (yes vs no); average monthly income classified using interquartile range ( IQR) as (< 1500, 1500-2499, 2500-3999, ≥ 4000 Birr); maternal anemia when the hemoglobin level was below 11 g/dl [46] and parity as (nullipara, primipara, multipara). Similarly, mid-upper arm circumference (MUAC) is known to be relatively stable during the course of pregnancy and is highly correlated to pre-pregnancy BMI [47, 48]. Women were categorized according to MUAC where < 28 cm was considered as normal and ≥ 28 cm taken to indicate pre-gestational overweight and/or obesity [49]. Mothers who drank coffee and alcohol daily or sometimes in a week after pregnancy were labeled as having exposure to coffee and alcohol, respectively.

Physical activities in the past one week were assessed using the short form of the International Physical Activity Questionnaire (IPAQ) [35]. Then, data was reported as metabolic equivalents according to IPAQ scoring protocol which categorized women into high, moderate and low groups [50, 51]. Likewise, a woman’s minimum dietary diversity was assessed by using the Food and Nutrition Technical Assistance (FANTA) 2016 and ten standardized lists of food items consumed day and night in the past 24 hours. The minimum dietary diversity score (MDDS) was dichotomized and coded as 0 and 1 for respondents who consumed less than five group items and greater than or equal to five items, respectively. Finally, the MDDS was categorized as adequate dietary diversity if the woman consumed five and more food item [34]. Antenatal depression was measured by using the Edinburgh Postnatal Depression Scale (EPDS) screening tool having ten specific questions with four Likert scale response options (most of the time, sometimes, not often, never), scored from 0 to 3 (a higher score indicating more depressive symptoms). We used a cut of point of 13 and above on the scale to identify women with depression [52, 53].

### Statistical analysis

Data were entered using Epi Info version 7 and analyzed using Stata 14 software. Descriptive statistics (frequencies, percentage, mean, and standard deviation (SD)) were used to describe participant characteristics. Pearson’s chi-square test was employed to compare categorical data between women with GDM and without GDM as well as to examine the distribution of independent variables and each adverse maternal outcome. Independent t-test was also used for the comparison of the mean difference of continuous variables.

Log-binomial model was used to determine the relative risk summary metric for the associations between GDM and adverse maternal outcomes and to control the effect of potential confounders. Separate log-binomial models were tested and presented for each outcome. Variables were included in the multivariable log-binomial model based on literature review and their association with each adverse maternal outcome (p-value ≤ 0.20) in the bivariate analysis. Crude relative risk (CRR) was generated in model I. In model II, the adjusted relative risk (ARR) for the associations between GDM and adverse maternal outcomes were determined after controlling for maternal and socio-demographic characteristics. In model III, in addition to model II confounding variables, it was adjusted for lifestyle variables (dietary diversity and physical activity). Model IV was adjusted for all confounding variables in models II and III plus antenatal depression. Variables in each model were mutually adjusted for each other. Moreover, multicollinearity between the variables was checked using the variance inflation factor (VIF). Finally, statistical significance was established at ARR≠ 1with a 95% CI and P-value ≤ 0.05.

## Results

### Characteristics of participants

**Fig. 1:**
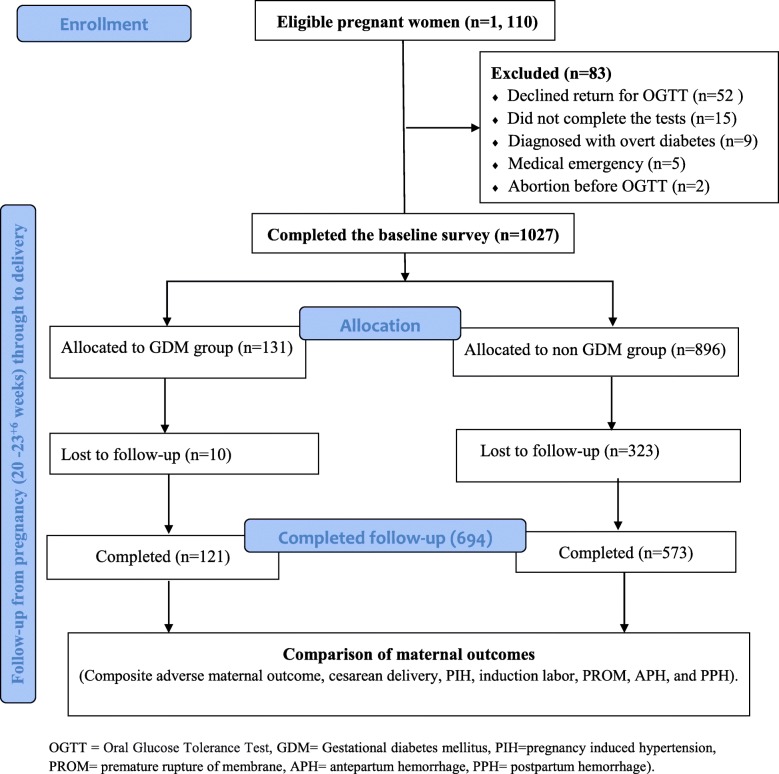
Study participant flow of the prospective cohort of pregnant women in Gondar town public health facilities, Northwest Ethiopia March 2018- March, 2019

A total of 1027 women were recruited at the ANC clinic and prospectively followed from pregnancy to delivery. Of those, 694 (67.6%) pregnant women completed the follow-up. Based on the GDM diagnosis criteria, 121 (17.4%) of the pregnant women had GDM, while 573 (82.6%) were without GDM **(**Fig. 1). The baseline characteristics of participants who completed the follow up was not statistically different from lost to follow-up participants (Table 1).

**Table 1:** Comparison between loss to follow-up and the available study participants at Gondar town public health facilities, Northwest Ethiopia: March, 2018-March, 2019 (*n*=1027)

Variables	Follow up status		*P* value
	Available n (%)	Lost to follow up n (%)	
Maternal age (years )			
< 25	197 (28.4)	126 (37.8)	0.001
25–29	241 (34.7)	125 (37.5)	
30–34	162 (23.3)	52 (15.6)	
≥ 35	94 (13.5)	30 (9)	
Marital status			
Married	639 (92.1)	306 (91.9)	0.903
Single and others^a^	55 (7.9)	27 (8.1)	
Educational level			
Not formal education	134 (19.3)	66 (19.8)	0.981
Primary education	155 (22.3)	74 (22.2)	
Secondary education and above	405 (58.4)	193 (58)	
Employment status			
Employed	275 (39.6)	131 (39.3)	0.946
Unemployed	419 (60.4)	202 (60.7)	
Monthly income (birr)			
< 1500	152 (21.9)	84 (25.2)	0.048
1500-2499	185 (26.7)	72 (21.6)	
2500-3999	165 (23.8)	65 (19.5)	
≥ 4000	192 (27.7)	112 (33.6)	
MUAC			
MUAC < 28 cm	576 (83)	276 (82.9)	0.964
MUAC ≥ 28 cm	118 (17)	57 (17.1)	
Anemic status^b^			
Normal	593 (87.1)	281 (85.7)	0.539
Anemia	88 (12.9)	47 (14.3)	
Parity			
Nullipara	340 (49)	164 (49.2)	0.373
Primipara	180 (25.9)	97 (29.1)	
Multipara	174 (25.1)	72 (21.6)	
Previous history of adverse pregnancy outcome^c^			
Yes	151 (37.8)	67 (35.6)	0.621
No	249 (62.3)	121 (64.4)	
Family history of diabetes			
Yes	50 (7.2)	12 (3.6)	0.023
No	644 (92.8)	321 (96.4)	
Alcohol use			
Yes	312 (45)	141 (42.3)	0.430
No	382 (55)	192 (57.7)	
Coffee intake			
Yes	484 (69.7)	252 (75.7)	0.048
No	210 (30.3)	81 (24.3)	
Dietary diversity status			
Adequate	322 (46.4)	175 (52.6)	0.065
Inadequate	372 (53.6)	158 (47.4)	
Level of physical activity			
High	208 (30.0)	114 (34.2)	0.387
Moderate	317 (45.7)	143 (42.9)	
Low	169 (24.4)	76 (22.8)	
Antenatal depression			
Yes	70 (10.1)	19 (5.7)	0.020
No	624 (89.9)	314 (94.3)	

^a^=Divorced/widowed ^b^=18 participants were missed at base line (*n*=1009) ^c^=(*n*=588) MUAC=mid upper arm circumference cm=centimeter

From all women who completed the follow-up, the mean age of mothers with GDM and no GDM was 30.9 (SD ± 5.01) and 27 (SD ± 5.04) years, respectively. Nearly three-fourths (74.3%) of the women with GDM and 470 (82.1%) without GDM attended school. Twenty-one percent of the exposed (GDM) group and 4.2 % of mothers without GDM had family history of diabetes. Most of the non GDM mothers were nulliparous. A higher proportion of anemia, over weight and / or obesity were observed among women with GDM compared to women with normal glucose level. Moreover, a larger percentage (58.7%) of women with GDM reported low level of physical activities than women in normal glucose profile. Besides, 76% and 25% of women with GDM experienced inadequate dietary diversity and antenatal depression symptoms, while 48.9% and 6.8% of women with normal glucose profile faced such problems, respectively (Table 2).

**Table 2:** Maternal, socio-demographic and life style characteristics of the study participants by GDM status among women completed the follow up from pregnancy through to delivery in Gondar town public health facilities, Northwest Ethiopia March 2018- March, 2019 (*n* = 694)

Variables	Total Participants (*n* = 694)	Blood glucose status		*P* value
		GDM n (%)	Non-GDM n (%)	
Maternal age (years )	27.69 ± 5.246	30.93 ± 5.015	27.01 ± 5.038	< 0.001
< 25	197 (28.4)	16 (13.2)	181 (31.6)	< 0.001
25–29	241 (34.7)	30 (24.8)	211 (36.8)	
30–34	162 (23.3)	42 (34.7)	120 (20.9)	
≥ 35	94 (13.5)	33 (27.3)	61 (10.6)	
Marital status				
Married	639 (92.1)	105 (86.8)	534 (93.2)	0.018
Single and others^a^	55 (7.9)	16 (13.2)	39 (6.8)	
Educational level				
Not formal education	134 (19.3)	31 (25.6)	103 (18)	0.032
Primary education	155 (22.3)	32 (26.4)	123 (21.5)	
Secondary education and above	405 (58.4)	58 (47.9)	347 (60.6)	
Employment status				
Employed	275 (39.6)	62 (51.2)	213 (37.2)	0.004
Unemployed	419 (60.4)	59 (48.8)	360 (62.8)	
Monthly income (birr)	3169.22 ± 3167.781	3232.96 ± 3197.301	3155.76 ± 3164.162	0.808
< 1500	152 (21.9)	29 (24)	123 (21.5)	0.472
1500-2499	185 (26.7)	26 (21.5)	159 (27.7)	
2500-3999	165 (23.8)	28 (23.1)	137(23.9)	
≥ 4000	192 (27.7)	38 (31.4)	154 (26.9)	
Parity				
Nullipara	340 (49)	49 (40.5)	291 (50.8)	0.052
Primipara	180 (25.9)	32 (26.4)	148 (25.8)	
Multipara	174 (25.1)	40 (33.1)	134 (23.4)	
Previous history of adverse pregnancy outcome***				
Yes	151 (37.8)	36 (45.6)	115 (35.8)	0.109
No	249 (62.3)	43 (54.4)	206 (64.2)	
Family history of diabetes				
Yes	50 (7.2)	26 (21.5)	24 (4.2)	< 0.001
No	644 (92.8)	95 (78.5)	549 (95.8)	
MUAC	24.74 ± 3.139	26.64 ± 4.074	24.34 ± 2.745	< 0.001
MUAC < 28 cm	576 (83)	76 (62.8)	500 (87.3)	< 0.001
MUAC ≥ 28 cm	118 (17)	45 (37.2)	73 (12.7)	
Hemoglobin (g/dl) ^b^	12.666 ±1.7575	12.333 ±1.7834	12.735 ±1.7455	0.024
Anemic status				
Normal	593 (87.1)	96 (81.4)	497 (88.3)	0.042
Anemia	88 (12.9)	22 (18.6)	66 (11.7)	
Alcohol use				
Yes	312 (45)	48 (39.7)	264 (46.1)	0.198
No	382 (55)	73 (60.3)	309 (53.9)	
Coffee intake				
Yes	484 (69.7)	82 (67.8)	402 (70.2)	0.603
No	210 (30.3)	39 (32.2)	171 (29.8)	
Dietary diversity status				
Adequate	322 (46.4)	29 (24)	293 (51.1)	< 0.001
Inadequate	372 (53.6)	92 (76)	280 (48.9)	
Level of physical activity				
High	208 (30)	20 (16.5)	188 (32.8)	< 0.001
Moderate	317 (45.7)	30 (24.8)	287 (50.1)	
Low	169 (24.4)	71 (58.7)	98 (17.1)	
Antenatal depression				
Yes	70 (10.1)	31 (25.6)	39 (6.8)	< 0.001
No	624 (89.9)	90 (74.4)	534 (93.2)	

*GDM* Gestational diabetes mellitus ^a^=Divorced or widowed ^b^ = participants with hemoglobin data (*n*=681)

^c^= participants with prior history of pregnancy (*n*=400) MUAC=mid upper arm circumference cm=centimeter

### Incidence of adverse maternal outcomes

**Fig. 2:**
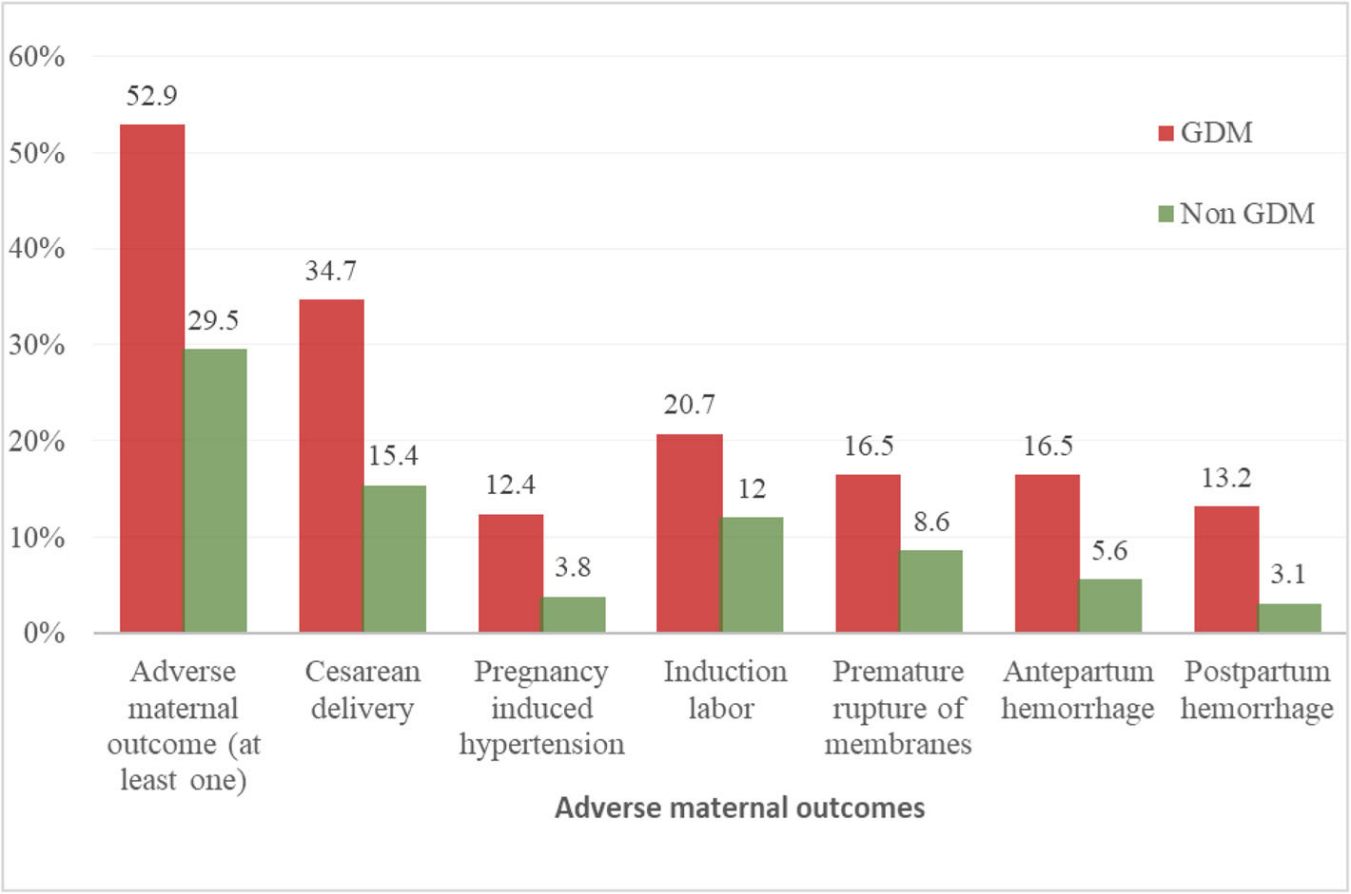
Adverse maternal outcomes among mothers with and without GDM among women completed the follow up from pregnancy through to delivery in Gondar town public health facilities, Northwest Ethiopia March 2018- March, 2019 (*n* = 694)

Of the total 694 women, 233 (33.6%) (95% CI: 30.5, 37.0) had at least one type of adverse maternal outcome. The proportion of adverse maternal outcome among mothers with and without GDM was 52.9% and 29.5%, respectively. The overall incidence of cesarean delivery was 18% (95% CI: 15.6, 21.8), PIH was 5.3% (95% CI: 3.7, 7.1), induction of labor was 13.5% (95% CI: 11, 16.1), PROM was 9.9% (95% CI: 7.9, 12.2), APH was 7.5% ( 95% CI: 5.5, 9.5), and PPH was 4.9% ( 95% CI: 3.3, 6.5). The incidence of cesarean delivery, PIH, induction of labor, PROM, APH, and PPH was higher among women with GDM compared to those with non- GDM **(**Fig. 2).

### Association of gestational diabetes mellitus with risk of adverse maternal outcomes

The results of the unadjusted log-binomial model (Model I) showed that maternal age, antenatal depression, employment status (marginally) and GDM were associated with composite adverse maternal outcome (at least one type). In multivariate model II, after adjusting for maternal and socio-demographic variables (maternal age, educational status, employment status, MUAC and GDM), the result showed that only GDM had significant association with composite adverse maternal outcome. We also examined whether the association between maternal and socio-demographic variables and GDM was mediated by the presence of lifestyle parameters (dietary diversity and physical activity) and run a separate multivariate model III with composite adverse maternal outcome. Still GDM was a key risk factor for composite adverse maternal outcome. After controlling for the effects of maternal and socio-demographic characteristics, lifestyle factors (dietary diversity and physical activity), and antenatal depression (Model IV), the association between GDM and composite adverse maternal outcome remained significant (ARR=1.58; 95% CI: 1.22, 2.04**)** (Table 3).

**Table 3:** Log-binomial regression analysis (models I–IV) showing the effect of gestational diabetes mellitus on composite adverse maternal outcomes among women completed the follow up from pregnancy through to delivery in Gondar town public health facilities, Northwest Ethiopia March 2018- March, 2019 (*n* = 694)

Variables	Composite adverse maternal outcome		Model I	Model II	Model III	Model IV
	Yes, n (%)	No, n (%)	CRR (95% CI)	ARR (95% CI)	ARR (95% CI)	ARR (95% CI)
GDM						
Yes	64 (52.9)	57 (47.1)	1.79 (1.45, 2.21) ^***^	1.71 (1.36, 2.14) ^***^	1.70 (1.33, 2.17) ^***^	1.58 (1.22, 2.04)^***^
No	169 (29.5)	404 (70.5)	1	1	1	1
Maternal age (years )						
< 25	54 (27.4)	143 (72.6)	1	1	1	1
25–29	82 (34)	159 (66)	1.24 (0.93, 1.65)	1.24 (0.94, 1.65)	1.24 (0.94, 1.65)	1.23 (0.93, 1.63)
30–34	59 (36.4)	103 (63.6)	1.33 (0.98, 1.80)	1.22 (0.89, 1.67)	1.22 (0.90, 1.67)	1.23 (0.90, 1.68)
≥ 35	38 (40.4)	56 (59.6)	1.47 (1.06, 2.06) *	1.32 (0.94, 1.85)	1.32 (0.94, 1.85)	1.35 (0.96, 1.89)
Educational level						
Not formal education	37 (27.6)	97 (72.4)	1	1	1	1
Primary education	54 (34.8)	101 (65.2)	1.26 (0.89, 1.79)	1.33 (0.94, 1.87)	1.33 (0.94, 1.88)	1.34 (0.95, 1.88)
Secondary education and above	142 (35.1)	263 (64.9)	1.27 (0.94, 1.72)	1.32 (0.96, 1.80)	1.32 (0.96, 1.81)	1.31 (0.96, 1.80)
Employment status						
Employed	104 (37.8)	171 (62.2)	1.23 (1.00, 1.51)	1.07 (0.86, 1.34)	1.08 (0.86, 1.34)	1.09 (0.88, 1.36)
Unemployed	129 (30.8)	290 (69.2)	1	1		1
MUAC						
MUAC < 28 cm	187 (32.5)	389 (67.5)	1	1	1	1
MUAC ≥ 28 cm	46 (39)	72 (61)	1.20 (0.93, 1.55)	1.05 (0.82, 1.36)	1.06 (0.82, 1.37)	1.05 (0.81, 1.36)
Dietary diversity status						
Adequate	103 (32)	219 (68)	1		1	1
Inadequate	130 (34.9)	242 (65.1)	1.09 (0.88, 1.35)		1.01 (0.82, 1.25)	0.94 (0.83, 1.27)
Level of physical activity						
High	66 (31.7)	142 (68.3)	1		1	1
Moderate	101(31.9)	216 (68.1)	1.01 (0.78,1.30)		0.95 (0.74, 1.23)	0.94 (0.73, 1.21)
Low	66 (39.1)	103 (60.9)	1.23 (0.94, 1.62)		0.98 (0.73, 1.30)	1.00 (0.75, 1.33)
Antenatal depression						
Yes	34 (48.6)	36 (51.4)	1.52 (1.17, 1.99) ^**^			1.28 (0.97, 1.68)
No	199 (31.9)	425 (68.1)	1			1

*= *P* < 0.05 **=*P* <0.01 ***= *P*< 0.001 1=Reference *CRR* Crude relative risk, *ARR* Adjusted relative risk, *CI* Confidence interval, *GDM* Gestational diabetes mellitus *MUAC* Mid-upper arm circumference

Notes: Model I: shows crude relative risk. Model II: Adjusted for maternal and socio-demographic variables (adjusted by maternal age, educational status, employment status, MUAC) Model III: Adjusted for maternal/socio-demographic variables plus life style parameters (physical activity and dietary diversity) Model IV: Adjusted for maternal/socio demographic variables, life style variables plus antenatal depression.

Similarly, separate log-binomial models were employed to identify the independent risk of GDM on cesarean delivery, PIH, induction of labor, PROM, APH, and PPH **(**see Additional file 1: Table S1-S6). Overall, after controlling the effects of maternal and socio-demographic characteristics, lifestyle parameters (physical activity and dietary diversity) including antenatal depression (Model IV), the risks of cesarean delivery were higher among women with GDM (ARR=1.67; 95% CI: 1.15, 2.44), PIH (ARR= 3.32; 95% CI: 1.55, 7.11), PROM (ARR= 1.83; 95% CI: 1.02, 3.27), APH (ARR= 2.10; 95% CI: 1.11, 3.98), PPH (ARR= 4.85; 95% CI:2.28, 10.30) compared to those without GDM. In this analysis, GDM was not significant risk for induction of labor (ARR= 1.20; 95% CI: 0.73, 1.98) (Table 4).

**Table 4:** Summary of Log-binomial regression analysis (models I–IV) showing the effect GDM on adverse maternal outcomes (each type) among women completed the follow up from pregnancy through to delivery in Gondar town public health facilities, Northwest Ethiopia March 2018- March, 2019 (*n* = 694)

Maternal outcome	Model I		Model II		Model III		Model IV	
	CRR (95% CI)	*P* -value	ARR (95% CI)	*P* value	ARR (95% CI)	*P* value	ARR (95% CI)	*P* value
Composite adverse maternal outcome	1.79 (1.45, 2.21)	< 0.001	1.71 (1.36, 2.14)	< 0.001	1.70 (1.33, 2.17)	< 0.001	1.58 (1.22, 2.04)	0.001
Cesarean delivery	2.26 (1.66, 3.08)	< 0.001	2.30 (1.59,3.34)	< 0.001	2.29 (1.50, 3.50)	< 0.001	1.67 (1.15, 2.44)	0.007
Pregnancy induced hypertension	3.23 (1.73, 6.04)	< 0.001	3.19 (1.67, 6.11)	0.000	2.99 (1.44, 6.20)	0.003	3.32 (1.55, 7.11)	0.002
Labor induction	1.72 (1.13, 2.59)	0.010	1.52 (0.97, 2.38)	0.065	1.40 (0.86, 2.28)	0.175	1.20 (0.73,1.98)	0.478
Premature rupture of membranes	1.93 (1.19, 3.13)	0.007	1.92 (1.15, 3.22)	0.013	1.86 (1.05, 3.27)	0.033	1.83 (1.02, 3.27)	0.043
Antepartum hemorrhage	2.96 (1.75, 4.99)	< 0.001	2.91 (1.65, 5.12)	< 0.001	2.25 (1.20, 4.20)	0.011	2.10 (1.11, 3.98)	0.022
Postpartum hemorrhage	4.21 (2.21, 8.02)	< 0.001	4.69 (2.47, 8.87)	< 0.001	4.34 (2.06, 9.13)	< 0.001	4.85 (2.28, 10.30)	< 0.001

*CRR* Crude relative risk, *ARR* Adjusted relative risk, *CI* Confidence interval

Notes: Model I: shows crude relative risk. Model II: Adjusted for maternal and socio-demographic variables. Model III: Adjusted for maternal/socio-demographic variables plus life style variables (dietary diversity and physical activity). Model IV: Adjusted for maternal/socio demographic variables, life style variables (dietary diversity and physical activity) plus antenatal depression.

## Discussion

This study was a prospective analysis of 694 women followed from pregnancy to delivery at selected public health facilities of Gondar town, Northwest Ethiopia. We compared the incidence of adverse maternal outcomes on women with and without GDM and examined the independent effects of GDM on adverse maternal outcomes.

The incidence of composite adverse maternal outcome was higher (52.9%) among women with GDM compared with women without GDM (8.1%). Specifically, the incidence of caesarean delivery, PIH, PROM, induction of labor, APH, and PPH was higher among women with GDM than those without GDM. This indicated that GDM can result a higher maternal morbidity. The findings were consistent with those of studies conducted in China [54], Qatar [24], Australia [55], and a review article on LMICs [2].

The multivariable log-binomial model showed that women with GDM had a higher risk for developing the composite adverse maternal outcome by 58% compared to women who had normal glucose profile. This is in line with studies in East Ethiopia [9], Uganda [56], Zimbabwe [57], Canada [17], and Saudi Arabia [58]. So, we can deduce that GDM is a serious problem with a significant impact on maternal outcomes. Thus, there is need to improve access to standard intervention measures to alleviate the plight of women with GDM.

We found the risk of cesarean delivery was higher among pregnant women with GDM than women without GDM by 67%. Previous studies [59–61] also demonstrated that the risk for cesarean delivery was high among GDM patients. For instance, GDM increased the incidence of cesarean sections (CS) from 30% [13] to 35% [62]. Though GDM alone is not an indication for CS before 38 weeks of gestation, it becomes evident that CS is a priority choice for many obstetricians due to different maternal and fetal complications arising from GDM [15]. Despite evidences showing the benefits of vaginal delivery, CS has been preferred for most diabetic pregnant women with previous operations for fear of the rupture of the uterus that may be associated with the risk of fetal macrosomia [63, 64]. Additionally, a study in Uganda [56] revealed that the modes of delivery were similar, but genital injuries were more common among women with GDM [56]. The primary indications of CS might be fetal macrosomia that resulted from GDM. On the other hand, CS can prevent poor obstetric outcomes and be a life-saving procedure for both the mother and the fetus [65]. However, there is a growing concern about unnecessary CS that leads to risks for maternal morbidity, neonatal death and neonatal admissions into intensive care units [66].

Women with GDM were three times at high risk for PIH compared to their counter parts. The finding was consistent with several other studies [4–9, 18, 67] which reported that GDM increased the incidence of PIH. Similarly, another study done in Eastern Ethiopia revealed that mothers who had GDM were three times more likely to develop preeclampsia than women who had not [9]. The association might be due to the nature of co-existing mutual risk factors, such as obesity, advanced maternal age and family history of diabetes and hypertension [68]. To maintain stable blood glucose levels, β cells in the pancreas subsequently increase the production of insulin, which results in hyperinsulinemia [69]. Evidence suggests that insulin resistance contribute to sodium retention and vasoconstriction in the pathogenesis of hypertensive disorders during pregnancy [70–73]. A better understanding of the association between these conditions may lead to implement more effective strategies on mutual risk factors during prenatal care.

Although the incidence of induction of labor was higher (20.7%) among women with GDM than women with normal glucose profile (12%), the adjusted analysis (model IV) showed no significant difference (ARR= 1.20; 95% CI: 0.73, 1.98). This is in line with a study conducted in Australia [55]. Evidence revealed that induction of labor was advised for women who had GDM to decrease further complication associated during delivery [74, 75]. On the other hand, this study identified antenatal depression increased the risk for the induction of labor by nearly two folds (ARR= 1.85; 95% CI: 1.15, 2.98). This finding suggests majority of induced labor perhaps due to stress. Studies also indicated that some healthcare providers encouraged women to elective induction due to the fear of complications of GDM [76–79]. Hence, psychosocial interventions were recommended to antenatal depression and subsequently reduced the risk of induction of labor.

This study also identified that mothers with GDM had more risk for PROM compared to women without GDM. Other studies demonstrated that there was a direct relationship between GDM and PROM [9, 80–83]. This might be due to the secondary complications of polyhydraminos and macrosomic babies caused by GDM and leads the head of the fetus to be arrested at the pelvic inlet, and the entire force exerted by the uterus is directed to the portion of membranes in contact with the internal os. Thus, early rupture of membranes is more likely to occur [23, 84].

In addition, this study found that the risk of APH was two times higher among women who had GDM than among those without GDM. The finding was in line with studies conducted in Ethiopia [9] and Qatar [24]. It might be because GDM has a negative effect on placenta previa and abruption placentae leading to APH. Similarly, we noted that women with GDM were nearly five times at increased risk for PPH than women without GDM. Previous studies [85–87] indicated that there was a strong association between GDM and PPH. This might be due to complications of GDM, such as fetal macrosoma or large for gestational age, shoulder dystocia, birth trauma and operative deliveries subsequently increased the risk for PPH [88]. The study suggested that due attention should be given to the management of obstetric hemorrhage among women with GDM.

The strengths of our study included its prospective nature and using the updated diagnostic criteria of GDM. However, several limitations have been noted. Firstly, it has significant drop-out rate or loss to follow-up. Secondly, the evidence does not show the difference in risks of adverse maternal outcomes based on the types of GDM treatment. Thirdly, stratified analysis was no conducted based on the time of exposure due to very small cases identified at late gestational age.

## Conclusions

The incidence of adverse maternal outcomes was higher among women with GDM than and among those without GDM. After controlling the confounders, GDM independently increased the risk of composite adverse maternal outcome, caesarean delivery, pregnancy induced hypertension, premature rupture of membranes, antepartum hemorrhage, and postpartum hemorrhage. This indicates that GDM is a serious problem with a great impact on pregnancy outcomes. Thus, we recommend enhancements of maternal care and intervention strategies for women with GDM to reduce these life-threatening obstetric complications and improve maternal outcomes. Further studies on the treatment, long-term and inter-generational effects of GDM are suggested.

## Supplementary information

**Additional file 1: Table S1.** Log-binomial regression analysis (models I–IV) showing the effect of gestational diabetes mellitus on cesarean delivery among women completed the follow up from pregnancy through to delivery in Gondar town public health facilities, Northwest Ethiopia March 2018- March, 2019 (*n* = 694). **Table S2.** Log-binomial regression analysis (models I–IV) showing the effect of gestational diabetes mellitus on pregnancy induced hypertension (PIH) among women completed the follow up from pregnancy through to delivery in Gondar town public health facilities, Northwest Ethiopia March 2018- March, 2019 (*n* = 694). **Table S3.** Log-binomial regression analysis (models I–IV) showing the effect of gestational diabetes mellitus on labor induction among women completed the follow up from pregnancy through to delivery in Gondar town public health facilities, Northwest Ethiopia March 2018- March, 2019 (*n*= 694). **Table S4.** Log-binomial regression analysis (models I–IV) showing the effect of gestational diabetes mellitus on premature rupture of membranes (PROM) among women completed the follow up from pregnancy through to delivery in Gondar town public health facilities, Northwest Ethiopia March 2018- March, 2019 (*n* = 694). **Table S5.** Log-binomial regression analysis (models I–IV) showing the effect of gestational diabetes mellitus on antepartum hemorrhage (APH) among women completed the follow up from pregnancy through to delivery in Gondar town public health facilities, Northwest Ethiopia March 2018- March, 2019 (*n* = 694). **Table S6.** Log-binomial regression analysis (models I–IV) showing the effect of gestational diabetes mellitus on postpartum hemorrhage (PPH) among women completed the follow up from pregnancy through to delivery in Gondar town public health facilities, Northwest Ethiopia March 2018- March, 2019 (*n*= 694).

## Data Availability

The datasets used and/or analyzed during the current study are available from the corresponding author on reasonable request.

## Abbreviations

APH: Antepartum hemorrhage
ARR: Adjusted relative risk
CI: Confidence interval
CS: Caesarean section
EDPS: Edinburgh postnatal depression scale
FANTA: Food and nutrition technical assistance
FPG: Fasting plasma glucose
GDM: Gestational diabetes mellitus
IPAQ: International physical activity questionnaire
LMIC: Low and middle-income countries
MDDS: Minimum dietary diversity score
MUAC: Mid-upper arm circumference
OGTT: Oral glucose tolerance test
PIH: Pregnancy induced hypertension
PPH: Postpartum hemorrhage
PROM: Premature rupture of membranes
WHO: World health organization
