# Supplementary material for: Effects of gestational diabetes mellitus on risk of adverse maternal outcomes: a prospective cohort study in Northwest Ethiopia
Source: BMC Pregnancy Childbirth. 2020 Feb 3;20:73. doi: 10.1186/s12884-020-2759-8 (PMC6998275; doi:10.1186/s12884-020-2759-8)
Supplement: Supplementary file 1 — Additional file 1: Table S1. Log-binomial regression analysis (models I–IV) showing the effect of gestational diabetes mellitus on cesarean delivery among women completed the follow up from pregnancy through to delivery in Gondar town public health facilities, Northwest Ethiopia March 2018- March, 2019 (n = 694). Table S2. Log-binomial regression analysis (models I–IV) showing the effect of gestational diabetes mellitus on pregnancy induced hypertension (PIH) among women completed the follow up from pregnancy through to delivery in Gondar town public health facilities, Northwest Ethiopia March 2018- March, 2019 (n = 694). Table S3. Log-binomial regression analysis (models I–IV) showing the effect of gestational diabetes mellitus on labor induction among women completed the follow up from pregnancy through to delivery in Gondar town public health facilities, Northwest Ethiopia March 2018- March, 2019 (n= 694). Table S4. Log-binomial regression analysis (models I–IV) showing the effect of gestational diabetes mellitus on premature rupture of membranes (PROM) among women completed the follow up from pregnancy through to delivery in Gondar town public health facilities, Northwest Ethiopia March 2018- March, 2019 (n = 694). Table S5. Log-binomial regression analysis (models I–IV) showing the effect of gestational diabetes mellitus on antepartum hemorrhage (APH) among women completed the follow up from pregnancy through to delivery in Gondar town public health facilities, Northwest Ethiopia March 2018- March, 2019 (n = 694). Table S6. Log-binomial regression analysis (models I–IV) showing the effect of gestational diabetes mellitus on postpartum hemorrhage (PPH) among women completed the follow up from pregnancy through to delivery in Gondar town public health facilities, Northwest Ethiopia March 2018- March, 2019 (n= 694). [file 12884_2020_2759_MOESM1_ESM.docx]

**Table S1**. Log-binomial regression analysis (models I–IV) showing the effect of gestational diabetes mellitus on cesarean delivery among women completed the follow up from pregnancy through to delivery in Gondar town public health facilities, Northwest Ethiopia March 2018- March, 2019 (n = 694)

| **Variables** | **Cesarean delivery** | | **Model I** | **Model II** | **Model III** | **Model IV** |
| --- | --- | --- | --- | --- | --- | --- |
|  | **Yes, n (%)** | **No, n (%)** | **CRR (95% CI)** | **ARR (95% CI)** | **ARR (95% CI)** | **ARR (95% CI)** |
| **GDM** |  |  |  |  |  |  |
| Yes | 42 (34.7) | 79 (65.3) | 2.26 (1.66, 3.08) ^***^ | 2.30 (1.59,3.34) ^***^ | 2.29 (1.50, 3.50) ^***^ | 1.67 (1.15, 2.44) ^**^ |
| No | 88 (15.4) | 485 (84.6) | 1 | 1 | 1 | 1 |
| Maternal age (years ) |  |  |  |  |  |  |
| < 25 | 26 (13.2) | 171 (86.8) | 1 | 1 | 1 | 1 |
| 25–29 | 44 (18.3) | 197(81.7) | 1.38 (0.88, 2.16) | 1.19 (0.61, 2.34) | 1.19 (0.61, 2.35) | 1.30 (0.84, 2.02) |
| 30–34 | 34 (21) | 128 (79) | 1.59 (0.99, 2.53) | 1.12 (0.56, 2.19) | 1.12 (0.57, 2.22) | 1.35 (0.85, 2.14) |
| ≥ 35 | 26 (27.7) | 68 (72.3) | 2.09 (1.29, 3.40) * | 1.51 (0.76, 3.02) | 1.53 (0.76, 3.07) | 1.77 (1.09, 2.87) * |
| Previous cesarean delivery |  |  |  |  |  |  |
| Yes | 22 (28.6) | 55 (71.4) | 1.51 (0.99, 2.30) | 1.44 (0.96, 2.16) | 1.44 (0.96, 2.18) | 1.43 (0.95, 2.18) |
| No | 61(18.9) | 262 (81.1) | 1 | 1 | 1 | 1 |
| Dietary diversity status |  |  |  |  |  |  |
| Adequate | 55 (17.1) | 267 (82.9) | 1 |  | 1 | 1 |
| Inadequate | 75 (20.2) | 297 (79.8) | 1.18 (0.86, 1.62) |  | 0.96 (0.65, 1.41) | 1.04 (0.77, 1.42) |
| Level of physical activity |  |  |  |  |  |  |
| High | 36 (17.3) | 172 (82.7) | 1 |  | 1 | 1 |
| Moderate | 53 (16.7) | 264 (83.3) | 0.97 (0.66,1.42) |  | 0.87 (0.54, 1.41) | 0.89 (0.61, 1.31) |
| Low | 41 (24.3) | 128 (75.7) | 1.40 (0.94, 2.09) |  | 0.95 (0.58, 1.57) | 1.04 (0.69, 1.58) |
| Antenatal depression |  |  |  |  |  |  |
| Yes | 27 (38.6) | 43 (61.4) | 2.33 (1.66, 3.30) *** |  |  | 1.93 (1.34, 2.77) *** |
| No | 103 (16.5) | 521 (83.5) | 1 |  |  | 1 |

*= P < 0.05 **=P <0.01 ***= P< 0.001 1=Reference CRR= crude relative risk ARR= adjusted relative risk CI=confidence interval GDM=gestational diabetes mellitus

**Notes**: **Model I:** shows relative risk. **Model II:** Adjusted for maternal and socio-demographic variables (adjusted by maternal age, previous cesarean delivery), **Model III:** Adjusted for maternal/socio-demographic variables plus life style parameters (physical activity and dietary diversity) **Model IV:** Adjusted for maternal/socio demographic variables, life style variables plus antenatal depression.

**Table S2.** Log-binomial regression analysis (models I–IV) showing the effect of gestational diabetes mellitus on pregnancy induced hypertension (PIH) among women completed the follow up from pregnancy through to delivery in Gondar town public health facilities, Northwest Ethiopia March 2018- March, 2019 (n = 694)

| **Variables** | **PIH** | | **Model I** | **Model II** | **Model III** | **Model IV** |
| --- | --- | --- | --- | --- | --- | --- |
|  | **Yes, n (%)** | **No, n (%)** | **CRR (95% CI)** | **ARR (95% CI)** | **ARR (95% CI)** | **ARR (95% CI)** |
| **GDM** |  |  |  |  |  |  |
| Yes | 15 (12.4) | 106 (87.6) | 3.23 (1.73, 6.04) ^***^ | 3.19 (1.67, 6.11) ^***^ | 2.99 (1.44, 6.20) ^**^ | **3.32 (1.55, 7.11)** ^**^ |
| No | 22 (3.8) | 551 (96.2) | 1 | 1 | 1 | 1 |
| Maternal age (years ) |  |  |  |  |  |  |
| < 25 | 6 (3.0) | 191 (97) | 1 | 1 | 1 | 1 |
| 25–29 | 15 (6.2) | 226(93.8) | 2.04 (0.81, 5.17) | 1.81 (0.71, 4.61) | 1.80 (0.71, 4.56) | 1.81 (0.71, 4.61) |
| 30–34 | 11 (6.8) | 151 (93.2) | 2.23 (0.84, 5.90) | 1.54 (0.56, 4.24) | 1.44 (0.52, 3.98) | 1.46 (0.53, 4.04) |
| ≥ 35 | 5 (5.3) | 89 (94.7) | 1.75 (0.55, 5.58) | 1.26 (0.36, 4.41) | 1.22 (0.35, 4.30) | 1.22 (0.35, 4.27) |
| Parity |  |  |  |  |  |  |
| Nullipara | 13 (3.8) | 327 (96.2) | 1 | 1 | 1 | 1 |
| Primipara | 15 (8.3) | 165 (91.7) | 2.18 (1.06, 4.48) * | 1.88 (0.91, 3.88) | 1.89 (0.91, 3.91) | 1.89 (0.91, 3.91) |
| Multipara | 9 (5.2) | 165 (94.8) | 1.35 (0.59, 3.10) | 1.09 (0.45, 2.64) | 1.13 (0.47, 2.71) | 1.09 (0.45, 2.61) |
| Dietary diversity status |  |  |  |  |  |  |
| Adequate | 14 (4.3) | 308 (95.7) | 1 |  | 1 | 1 |
| Inadequate | 23 (6.2) | 349 (93.8) | 1.42 (0.74, 2.72) |  | 1.08 (0.56, 2.07) | 1.08 (0.56, 2.07) |
| Level of physical activity |  |  |  |  |  |  |
| High | 5 (2.4) | 203 (97.6) | 1 |  | 1 | 1 |
| Moderate | 18 (5.7) | 299 (94.3) | 2.36 (0.89,6.26) |  | 2.38 (0.90, 6.27) | 2.43 (0.92, 6.42) |
| Low | 14 (8.3) | 155 (91.7) | 3.45 (1.27, 9.37) * |  | 2.16 (0.77, 6.09) | 2.16 (0.77, 6.07) |
| Antenatal depression |  |  |  |  |  |  |
| Yes | 4 (5.7) | 66 (94.3) | 1.08 (0.39, 2.96) |  |  | 0.63 (0.23, 1.75) |
| No | 33 (5.3) | 591 (94.7) | 1 |  |  | 1 |

*= P < 0.05 **=P <0.01 ***= P< 0.001 1=Reference CRR= crude relative risk ARR= adjusted relative risk CI=confidence interval GDM=gestational diabetes mellitus PIH= pregnancy induced hypertension

**Notes: Model I:** shows relative risk. **Model II:** Adjusted for maternal and socio-demographic variables (adjusted by maternal age, parity) **Model III:** Adjusted for maternal/socio-demographic variables plus life style parameters (physical activity and dietary diversity) **Model IV:** Adjusted for maternal/socio demographic variables, life style variables plus antenatal depression.

**Table S3.** Log-binomial regression analysis (models I–IV) showing the effect of gestational diabetes mellitus on labor induction among women completed the follow up from pregnancy through to delivery in Gondar town public health facilities, Northwest Ethiopia March 2018- March, 2019 (n= 694)

| **Variables** | **Labor induction** | | **Model I** | **Model II** | **Model III** | **Model IV** |
| --- | --- | --- | --- | --- | --- | --- |
|  | **Yes, n (%)** | **No, n (%)** | **CRR (95% CI)** | **ARR (95% CI)** | **ARR (95% CI)** | **ARR (95% CI)** |
| **GDM** |  |  |  |  |  |  |
| Yes | 25 (20.7) | 96 (79.3) | 1.72 (1.13, 2.59) ^**^ | 1.52 (0.97, 2.38) | 1.40 (0.86, 2.28) | **1.20 (0.73,1.98)** |
| No | 69 (12) | 504 (88) | 1 | 1 | 1 | 1 |
| Maternal age (years ) |  |  |  |  |  |  |
| < 25 | 22 (11.2) | 175 (88.8) | 1 | 1 | 1 | 1 |
| 25–29 | 32 (13.3) | 209 (86.7) | 1.19 (0.71, 1.98) | 1.20 (0.72, 2.00) | 1.19 (0.71, 1.99) | 1.17 (0.70, 1.94) |
| 30–34 | 22 (13.6) | 140 (86.4) | 1.21 (0.70, 2.11) | 1.19 (0.66, 2.13) | 1.17 (0.65, 2.09) | 1.13 (0.63, 2.02) |
| ≥ 35 | 18 (19.1) | 76 (80.9) | 1.71 (0.97, 3.04) | 1.89 (0.99, 3.60) | 1.91 (1.01, 2.80) ^*^ | 1.97 (1.03, 3.74) ^*^ |
| Parity |  |  |  |  |  |  |
| Nullipara | 44 (12.9) | 296 (87.1) | 1 | 1 | 1 | 1 |
| Primipara | 30 (16.7) | 150 (83.3) | 1.29 (0.84, 1.97) | 1.25 (0.80, 1.94) | 1.24 (0.80, 1.93) | 1.21 (0.78, 1.88) |
| Multipara | 20 (11.5) | 154 (88.5) | 0.89 (0.54, 1.46) | 0.67 (0.39, 1,17) | 0.67 (0.38, 1.16) | 0.68 (0.39, 1.19) |
| MUAC |  |  |  |  |  |  |
| MUAC < 28 cm | 73 (12.7) | 503 (87.3) | 1 | 1 | 1 | 1 |
| MUAC ≥ 28 cm | 21 (17.8) | 97 (82.2) | 1.40 (0.90, 2.19) | 1.24 (0.77, 1.98) | 1.21 (0.76, 1.94) | 1.15 (0.72, 1.86) |
| Dietary diversity status |  |  |  |  |  |  |
| Adequate | 41 (12.7) | 281 (87.3) | 1 |  | 1 | 1 |
| Inadequate | 53 (14.2) | 319 (85.8) | 1.12 (0.77, 1.64) |  | 1.01 (0.68, 1.48) | 1.01(0.69, 1.48) |
| Level of physical activity |  |  |  |  |  |  |
| High | 23 (11.1) | 185 (88.9) | 1 |  | 1 | 1 |
| Moderate | 41 (12.9) | 276 (87.1) | 1.17 (0.72,1.89) |  | 1.15 (0.72, 1.86) | 1.12 (0.70, 1.81) |
| Low | 30 (17.8) | 139 (82.2) | 1.61 (0.97, 2.66) |  | 1.36 (0.80, 2.31) | 1.38 (0.82, 2.35) |
| Antenatal depression |  |  |  |  |  |  |
| Yes | 18 (25.7) | 52 (74.3) | 2.11 (1.35, 3.31) ^***^ |  |  | 1.85 (1.15, 2.98) ^*^ |
| No | 76 (12.2) | 548 (87.8) | 1 |  |  | 1 |

*= P < 0.05 **=P <0.01 ***= P< 0.001 1=Reference CRR= crude relative risk ARR= adjusted relative risk CI=confidence interval GDM=gestational diabetes mellitus MUAC= mid-upper arm circumference

**Notes: Model I:** shows relative risk. **Model II:** Adjusted for maternal and socio-demographic variables (adjusted by maternal age, parity, MUAC); **Model III:** Adjusted for maternal/socio-demographic variables plus life style parameters (physical activity and dietary diversity) **Model IV:** Adjusted for maternal/socio demographic variables, life style variables plus antenatal depression.

**Table S4.** Log-binomial regression analysis (models I–IV) showing the effect of gestational diabetes mellitus on premature rupture of membranes (PROM) among women completed the follow up from pregnancy through to delivery in Gondar town public health facilities, Northwest Ethiopia March 2018- March, 2019 (n = 694)

| **Variables** | **PROM** | | **Model I** | **Model II** | **Model III** | **Model IV** |
| --- | --- | --- | --- | --- | --- | --- |
|  | **Yes, n (%)** | **No, n (%)** | **CRR (95% CI)** | **ARR (95% CI)** | **ARR (95% CI)** | **ARR (95% CI)** |
| **GDM** |  |  |  |  |  |  |
| Yes | 20 (16.5) | 101 (83.5) | 1.93 (1.19, 3.13) ^**^ | 1.92 (1.15, 3.22) ^**^ | 1.86 (1.05, 3.27) ^*^ | **1.83 (1.02, 3.27)** **^*^** |
| No | 49 (8.6) | 524(91.4) | 1 | 1 | 1 | 1 |
| Maternal age (years ) |  |  |  |  |  |  |
| < 25 | 16 (8.1) | 181 (91.9) | 1 | 1 | 1 | 1 |
| 25–29 | 28 (11.6) | 213 (88.4) | 1.43 (0.80, 2.57) | 1.52 (0.84, 2.73) | 1.53 (0.85, 2.75) | 1.53 (0.85, 2.75) |
| 30–34 | 15 (9.3) | 147 (90.7) | 1.14 (0.58, 2.23) | 1.15 (0.56, 2.37) | 1.16 (0.57, 2.39) | 1.16 (0.56, 2.38) |
| ≥ 35 | 10 (10.6) | 84 (89.4) | 1.31 (0.62, 2.78) | 1.38 (0.61, 3.13) | 1.33 (0.59, 3.02) | 1.33 (0.59, 3.02) |
| Parity |  |  |  |  |  |  |
| Nullipara | 37 (10.9) | 303 (89.1) | 1 | 1 | 1 | 1 |
| Primipara | 19 (10.6) | 161 (89.4) | 0.97 (0.58, 1.64) | 0.86 (0.50, 1.47) | 0.87 (0.51, 1.48) | 0.87 (0.51, 1.48) |
| Multipara | 13 (7.5) | 161 (92.5) | 0.69 (0.37, 1.26) | 0.56 (0.29, 1.09) | 0.56 (0.29, 1.08) | 0.56 (0.29, 1.08) |
| Monthly income (birr) |  |  |  |  |  |  |
| < 1500 | 12 (7.9) | 140 (92.1) | 1 | 1 | 1 | 1 |
| 1500-2499 | 18 (9.7) | 167 (90.3) | 1.23 (0.61, 2.48) | 1.34 (0.67, 2.69) | 1.35 (0.67, 2.70) | 1.35 (0.68, 2.72) |
| 2500-3999 | 13 (7.9) | 152 (92.1) | 0.99 (0.47, 2.12) | 1.04 (0.49, 2.20) | 1.06 (0.50, 2.24) | 1.07 (0.50, 2.26) |
| ≥ 4000 | 26 (13.5) | 166 (86.5) | 1.72 (0.89, 3.29) | 1.77 (0.93, 3.36) | 1.78 (0.93, 3.38) | 1.79 (0.94, 3.41) |
| MUAC |  |  |  |  |  |  |
| MUAC < 28 cm | 54 (9.4) | 522 (90.6) | 1 | 1 | 1 | 1 |
| MUAC ≥ 28 cm | 15 (12.7) | 103 (87.3) | 1.36 (0.79, 2.32) | 1.25 (0.71, 2.20) | 1.23 (0.70, 2.18) | 1.23 (0.69, 2.17) |
| Dietary diversity status |  |  |  |  |  |  |
| Adequate | 24 (7.5) | 298 (82.5) | 1 |  | 1 | 1 |
| Inadequate | 45 (12.1) | 327 (87.9) | 1.62 (1.01, 2.60) ^*^ |  | 1.49 (0.92, 2.42) | 1.50 (0.92, 2.43) |
| Level of physical activity |  |  |  |  |  |  |
| High | 21 (10.1) | 187 (89.9) | 1 |  | 1 | 1 |
| Moderate | 30 (9.5) | 287 (90.5) | 0.94 (0.55,1.59) |  | 0.95 (0.56, 1.60) | 0.94 (0.56, 1.59) |
| Low | 18 (10.7) | 151 (89.3) | 1.05 (0.58, 1.91) |  | 0.82 (0.44, 1.51) | 0.81 (0.44, 1.51) |
| Antenatal depression |  |  |  |  |  |  |
| Yes | 9 (12.9) | 61 (87.1) | 1.34 (0.69, 2.58) |  |  | 1.08 (0.55, 2.13) |
| No | 60 (9.6) | 564 (90.4) | 1 |  |  | 1 |

*= P < 0.05 **=P <0.01 ***= P< 0.001 1=Reference CRR= crude relative risk ARR= adjusted relative risk CI=confidence interval GDM=gestational diabetes mellitus PROM=premature rupture of membranes MUAC= mid-upper arm circumference

**Notes: Model I:** shows crude odds ratios. **Model II:** Adjusted for maternal and socio-demographic variables (adjusted by maternal age, income, parity, MUAC); **Model III:** Adjusted for maternal/socio-demographic variables plus life style parameters (physical activity and dietary diversity) **Model IV:** Adjusted for maternal/socio demographic variables, life style variables plus antenatal depression.

**Table S5**. Log-binomial regression analysis (models I–IV) showing the effect of gestational diabetes mellitus on antepartum hemorrhage (APH) among women completed the follow up from pregnancy through to delivery in Gondar town public health facilities, Northwest Ethiopia March 2018- March, 2019 (n = 694)

| **Variables** | **APH** | | **Model I** | **Model II** | **Model III** | **Model IV** |
| --- | --- | --- | --- | --- | --- | --- |
|  | **Yes, n (%)** | **No, n (%)** | **CRR (95% CI)** | **ARR (95% CI)** | **ARR (95% CI)** | **ARR (95% CI)** |
| **GDM** |  |  |  |  |  |  |
| Yes | 20 (16.5) | 101 (83.5) | 2.96 (1.75, 4.99) ^***^ | 2.91 (1.65, 5.12) ^***^ | 2.25 (1.20, 4.20) ^*^ | **2.10 (1.11, 3.98)** ^*^ |
| No | 32 (5.6) | 541 (94.4) | 1 | 1 | 1 | 1 |
| Maternal age (years ) |  |  |  |  |  |  |
| < 25 | 12 (6.1) | 185 (93.9) | 1 | 1 | 1 | 1 |
| 25–29 | 14 (5.8) | 227 (94.2) | 0.95 (0.45, 2.01) | 0.89 (0.42, 1.87) | 0.91 (0.44, 1.91) | 0.90 (0.43, 1.88) |
| 30–34 | 16 (9.9) | 146 (90.1) | 1.62 (0.79, 3.33) | 1.23 (0.60, 2.55) | 1.29 (0.63, 2.65) | 1.26 (0.62, 2.57) |
| ≥ 35 | 10 (10.6) | 84 (89.4) | 1.75 (0.78, 3.90) | 1.11(0.49, 2.54) | 1.15 (0.51, 2.59) | 1.22 (0.54, 2.74) |
| Educational level |  |  |  |  |  |  |
| Not formal education | 12 (9) | 122 (91) | 1 | 1 | 1 | 1 |
| Primary education | 8 (5.2) | 147 (94.8) | 0.58 (0.24, 1.37) | 0.59 (0.25, 1.38) | 0.57 (0.24, 1.33) | 0.56 (0.24, 1.32) |
| Secondary education and above | 32 (7.9) | 373 (92.1) | 0.88 (0.47, 1.66) | 0.98 (0.51, 1.86) | 0.95 (0.50, 1.81) | 0.98 (0.51, 1.87) |
| Monthly income (birr) |  |  |  |  |  |  |
| < 1500 | 6 (3.9) | 146 (96.1) | 1 | 1 | 1 | 1 |
| 1500-2499 | 18 (9.7) | 167 (90.3) | 2.46 (1.003, 6.05) ^*^ | 2.64 (1.09, 6.42) ^*^ | 2.52 (1.04, 6.13) ^*^ | 2.58 (1.06, 6.27) ^*^ |
| 2500-3999 | 10 (6.1) | 155 (93.9) | 1.54 (0.57, 4.12) | 1.57 (0.59, 4.20) | 1.60 (0.60, 4.29) | 1.63 (0.61, 4.34) |
| ≥ 4000 | 18 (9.4) | 174 (90.6) | 2.38 (0.97 , 5.84) | 2.33 (0.95, 5.73) | 2.39 (0.97, 5.90) | 2.49 (1.01, 6.14) ^*^ |
| MUAC |  |  |  |  |  |  |
| MUAC < 28 cm | 40 (6.9) | 536 (93.1) | 1 | 1 | 1 | 1 |
| MUAC ≥ 28 cm | 12 (10.2) | 106 (89.8) | 1.46 (0.79, 2.71) | 1.05 (0.56, 1.97) | 0.99 (0.52, 1.87) | 0.94 (0.49, 1.79) |
| Dietary diversity status |  |  |  |  |  |  |
| Adequate | 17 (5.3) | 305 (94.7) | 1 |  | 1 | 1 |
| Inadequate | 35 (9.4) | 337 (90.6) | 1.78 (1.02, 3.12) ^*^ |  | 1.42 (0.80, 2.51) | 1.46 (0.83, 2.57) |
| Level of physical activity |  |  |  |  |  |  |
| High | 14 (6.7) | 194 (93.3) | 1 |  | 1 | 1 |
| Moderate | 16 (5) | 301 (95) | 0.75 (0.37,1.50) |  | 0.74 (0.37, 1.47) | 0.73 (0.37, 1.47) |
| Low | 22 (13) | 147 (87) | 1.93 (1.02, 3.66) ^*^ |  | 1.33 (0.67, 2.64) | 1.33 (0.67, 2.64) |
| Antenatal depression |  |  |  |  |  |  |
| Yes | 9 (12.9) | 61 (87.1) | 1.87 (0.95, 3.66) |  |  | 1.55 (0.77, 3.13) |
| No | 43 (6.9) | 581 (93.1) | 1 |  |  | 1 |

*= P < 0.05 **=P <0.01 ***= P< 0.001 1=Reference CRR= crude relative risk ARR= adjusted relative risk CI=confidence interval APH=antepartum hemorrhage GDM=gestational diabetes mellitus MUAC= mid-upper arm circumference

**Notes: Model I:** shows relative risk. **Model II:** Adjusted for maternal and socio-demographic variables (adjusted by maternal age, educational status, income, MUAC); **Model III:** Adjusted for maternal/socio-demographic variables plus life style parameters (physical activity and dietary diversity) **Model IV:** Adjusted for maternal/socio demographic variables, life style variables plus antenatal depression.

**Table S6.** Log-binomial regression analysis (models I–IV) showing the effect of gestational diabetes mellitus on postpartum hemorrhage (PPH) among women completed the follow up from pregnancy through to delivery in Gondar town public health facilities, Northwest Ethiopia March 2018- March, 2019 (n= 694)

| **Variables** | **PPH** | | **Model I** | **Model II** | **Model III** | **Model 4** |
| --- | --- | --- | --- | --- | --- | --- |
|  | **Yes, n (%)** | **No, n (%)** | **CRR (95% CI)** | **ARR (95% CI)** | **ARR (95% CI)** | **ARR (95% CI)** |
| **GDM** |  |  |  |  |  |  |
| Yes | 16 (13.2) | 105 (86.8) | 4.21 (2.21, 8.02) ^***^ | 4.69 (2.47, 8.87) ^***^ | 4.34 (2.06, 9.13) ^***^ | **4.85 (2.28, 10.30)** ^***^ |
| No | 18 (3.1) | 555 (96.9) | 1 | 1 | 1 | 1 |
| Monthly income (birr) |  |  |  |  |  |  |
| < 1500 | 5 (3.3) | 147 (96.7) | 1 | 1 | 1 | 1 |
| 1500-2499 | 7 (3.8) | 178 (96.2) | 1.15 (0.37, 3.55) | 1.34 (0.44,4.06) | 1.33(0.44,4.06) | 1.32 (0.44, 4.01) |
| 2500-3999 | 8 (4.8) | 157 (95.2) | 1.47 (0.49, 4.41) | 1.68 (0.57,4.93) | 1.68 (0.57,4.95) | 1.62 (0.55, 4.78) |
| ≥ 4000 | 14 (7.3) | 178 (92.7) | 2.22 (0.82, 6.02) | 2.49 (0.94, 6.60) | 2.37(0.89,6.33) | 2.23 (0.84, 5.92) |
| Parity |  |  |  |  |  |  |
| Nullipara | 22 (6.5) | 318 (93.5) | 1 | 1 | 1 | 1 |
| Primipara | 7 (3.9) | 173 (96.1) | 0.60 (0.26, 1.38) | 0.54 (0.24, 1.22) | 0.55 (0.24, 1.24) | 0.57 (0.25, 1.28) |
| Multipara | 5 (2.9) | 169 (97.1) | 0.44 (0.17, 1.15) | 0.35 (0.14, 0.89) ^*^ | 0.35 (0.14, 0.89) ^*^ | 0.35 (0.14, 0.88) ^*^ |
| Dietary diversity status |  |  |  |  |  |  |
| Adequate | 11 (3.4) | 311 (96.6) | 1 |  | 1 | 1 |
| Inadequate | 23 (6.2) | 349 (93.8) | 1.81 (0.90, 3.66) |  | 1.33 (0.63,2.79) | 1.29 (0.62, 2.70) |
| Level of physical activity |  |  |  |  |  |  |
| High | 11 (5.3) | 197 (94.7) | 1 |  | 1 | 1 |
| Moderate | 11 (3.5) | 306 (96.5) | 0.66 (0.29,1.49) |  | 0.69 (0.31, 1.54) | 0.75 (0.33, 1.68) |
| Low | 12 (7.1) | 157 (92.9) | 1.34 (0.61, 2.97) |  | 0.77(0.34, 1.74) | 0.80 (0.36, 1.77) |
| Antenatal depression |  |  |  |  |  |  |
| Yes | 2 (2.9) | 68 (97.1) | 0.56 (0.14, 2.28) |  |  | 0.38 (0.092,1.54) |
| No | 32 (5.1) | 592 (94.9) | 1 |  |  | 1 |

*= P < 0.05 **=P <0.01 ***= P< 0.001 1=Reference CRR= crude relative risk ARR= adjusted relative risk CI=confidence interval PPH= postpartum hemorrhage GDM=gestational diabetes mellitus

**Notes: Model I:** shows = crude relative risk. **Model II:** Adjusted for maternal and socio-demographic variables (adjusted by parity, income). **Model III:** Adjusted for maternal/socio-demographic variables plus life style parameters (physical activity and dietary diversity). **Model IV:** Adjusted for maternal/socio demographic variables, life style variables plus antenatal depression.
